# Natural Biostimulants Elicit Plant Immune System in an Integrated Management Strategy of the Postharvest Green Mold of Orange Fruits Incited by *Penicillium digitatum*

**DOI:** 10.3389/fpls.2021.684722

**Published:** 2021-06-15

**Authors:** Federico La Spada, Francesco Aloi, Maurizio Coniglione, Antonella Pane, Santa Olga Cacciola

**Affiliations:** ^1^Department of Agriculture, Food and Environment (Di3A), University of Catania, Catania, Italy; ^2^Department of Agricultural, Food and Forest Sciences, University of Palermo, Palermo, Italy; ^3^Decco Italia S.R.L., Belpasso, Italy

**Keywords:** biostimulants, resistance-inducers, algal and plant extracts, imazalil, *Citrus sinensis*, resistance genes, fungicide residues

## Abstract

This study was aimed at testing the integrated use of a natural biostimulant based on seaweed (*Ascophyllum nodosum*) and plant (alfalfa and sugarcane) extracts and reduced dosages of the conventional synthetic fungicide Imazalil (IMZ) to manage postharvest rots of orange fruits. The following aspects were investigated: (i) the effectiveness of postharvest treatment with natural biostimulant alone or in mixture with IMZ at a reduced dose against green mold caused by *Penicillium digitatum*; (ii) the differential expression of defense genes in orange fruits treated with the natural biostimulant both alone and in combination with a reduced dose of IMZ; (iii) the persistence of the inhibitory activity of both biostimulant and the mixture biostimulant/IMZ against green mold; and (iv) the residue level of fungicide in citrus peel when applied alone or in combination with the biostimulant. Treatments with the chemical plant resistance-inducer potassium phosphite, alone or with a reduced dose of IMZ, were included for comparison. The mixture of natural biostimulant and IMZ at a low dose consistently reduced the incidence and severity of fruit green mold and induced a significant increase of the expression level of β-1,3-glucanase-, peroxidase (PEROX)-, and phenylalanine ammonia-lyase (PAL)-encoding genes in fruit peel, suggesting that the natural biostimulant elicits a long-lasting resistance of citrus fruits to infections by *P. digitatum*. Interestingly, the residual concentration of IMZ in fruits treated with the biostimulant/fungicide mixture was significantly lower than that of IMZ in fruits treated only with the fungicide at the same dose and by far below the threshold values set by the European Union. This study laid the foundations for (i) conceiving a practical and more eco-friendly alternative to the conventional postharvest management of green mold of citrus fruits, based almost exclusively on the use of synthetic fungicide IMZ, alone or mixed with potassium phosphite and (ii) providing a better insight into the mechanisms of disease resistance induction by biostimulants.

## Introduction

Molds produce major postharvest losses of citrus fruits during storage, processing, transportation, and marketing (Eckert and Eaks, 1989). Green mold caused by *Penicillium digitatum* is the most damaging and widespread postharvest disease of citrus fruits worldwide (Ismail and Zhang, 2004; Youssef et al., 2014). The typical symptoms of the disease are a soft rot and a dense, green mass of conidia covering the peel. The ability of *P. digitatum* to produce a huge amount of conidia enables the inoculum of the pathogen to be widespread and contaminate citrus fruits in the field before harvest as well as in storage rooms, transit containers, packinghouses, boxes, and marketplaces (Ismail and Zhang, 2004). Infection takes place only through wounds where nutrients are available to stimulate spore germination, and fruit decay begins at these infected injury sites. At the early stages, the infection appears as a soft area surrounding the wound; rapidly, a white mycelium appears on the soft area and starts producing green conidia. The rapid infection progress leads, in a few days, to the complete decay of the fruits, which at the late stages appear to be completely covered by green conidia (Ismail and Zhang, 2004). Due to serious losses caused by the disease, the postharvest management of citrus green mold comprises specific practices to prevent it, such as washing and drenching with synthetic fungicides (Ismail and Zhang, 2004). Presently, Imazalil (IMZ) is the most commonly used fungicide for postharvest treatments of citrus fruits (Davé et al., 1989; Holmes and Eckert, 1999; Ismail and Zhang, 2004; Moraes Bazioli et al., 2019).

However, apart from toxicological risks due to the presence of residues in the fruit peel and the implications related to the disposal of washing water containing high levels of fungicide residues in packinghouses (Strange and Scott, 2005; Arslan, 2015; Altieri et al., 2016), a constraint of the postharvest management of this disease based on the intensive use of fungicides is the occurrence of resistant pathogen populations with a consequent reduction of the fungicide effectiveness (Davé et al., 1989; Eckert, 1990; Brent and Hollomon, 2007; Kanetis et al., 2007).

To minimize the risks resulting from the use of synthetic fungicides and in accordance with the European Directive 2009/128/EC, aimed at reducing the use of pesticides in agriculture, alternative and more eco-friendly strategies are being sought in the past few years (Stracquadanio et al., 2020). They include the reinforcement of natural plant defense mechanisms by eco-friendly external inputs. Plants can defend themselves against pathogens by detecting the invaders through the perception of signal molecules, also known as elicitors, often localized in the cell wall of the attacking organism (Cluzet et al., 2004; Khan et al., 2009; La Spada et al., 2021). Signal molecules include oligosaccharides and polysaccharides, peptides, proteins, and lipids (Boller, 1995; Côté et al., 1998). Elicitor perception triggers various signaling pathways followed by the synthesis of further signal molecules, such as salicylic acid, jasmonic acid, and ethylene, which are in turn activators of the synthesis of defense metabolites in the plants (Cluzet et al., 2004). An important category of induced plant defense metabolites is represented by pathogenesis-related (PR) proteins; among these, the PR-2 family enzyme β-1,3-glucanase can degrade the β-1,3-glucan, which is one of the main constituents of the fungal cell wall (Youssef et al., 2014; La Spada et al., 2020). Another family of PR-proteins playing a key role in several metabolic responses in plant defense processes are plant peroxidases; they are involved in the formation of auxin metabolism, lignin, and suberin, cross-linking of cell wall components, the synthesis of phytoalexin, and the metabolism of reactive oxygen species (ROS) and reactive nitrogen species (RNS; Almagro et al., 2009). A high concentration of peroxidases has been also found in citrus peels infected by *P. digitatum*, indicating that this family of metabolites plays a primary role in the resistance to citrus green mold (Ballester et al., 2006).

Apart from PR-proteins, the phenylpropanoid metabolism has also been associated with induced plant defenses during the pathogen infection process (Dixon and Paiva, 1995; Ballester et al., 2006). In citrus fruits, the phenylalanine ammonia-lyase (PAL) leads to the synthesis of flavonoids (Del Río et al., 1998; Arcas et al., 2000) and p-coumaric acid derivatives (Kim et al., 1991; Afek et al., 1999), which may help protect the fruit from pathogen attack. PAL activity in citrus fruits has been reported to be a consequence of physical or chemical stimuli such as UV (Droby et al., 2002) and gamma irradiations (Riov et al., 1968), heat (Martinez-Tellez and Lafuente, 1997), and ethylene (Riov et al., 1969) treatments.

During the last years, the use of plant biostimulants, including disease defense inducers, in agriculture is increasingly expanding (Calvo et al., 2014). A plant biostimulant is defined as any substance or microorganism applied to plants to enhance nutrition efficiency, abiotic stress tolerance, and/or crop quality traits, regardless of its content in nutrients (du Jardin, 2015). Regardless of the strict definition, some categories of plant biostimulants, such as seaweed extracts and plant derivatives, have proven their effectiveness in the elicitation of plant defense mechanisms against biotic stresses (du Jardin, 2015; Pylak et al., 2019). Extracts of green algae belonging to the *Ulva* genus elicited the synthesis of PR-proteins in *Medicago trunculata*, providing subsequent protection from *Colletotrichum trifolii* infection (Cluzet et al., 2004); foliar and root applications with a formulation of *Ascophyllum nodosum* increased the transcriptions of several defense-related genes, including β-1,3-glucanase, peroxidase (PEROX) and PAL in cucumber plants, resulting in a significant decrease in disease incidence as a consequence of the infection by *Phytophthora melonis* (Abkhoo and Sabbagh, 2016). Plant derivatives from alfalfa (*Medicago sativa*) enhanced both the PAL activity and flavonoid content, as well as the relative genes transcriptions in maize grown hydroponically (Ertani et al., 2013). Other plant derivatives employed as biostimulants include by-products from sugarcane transformation, such as molasses and vinasses, which in agriculture have been mainly used as bio-fertilizers (Christofoletti et al., 2013). During the last years, some studies started to investigate the possibility to control the *in vitro* development of soil-borne fungal pathogens at different concentrations of vinasses (Santos et al., 2009). However, there is a substantial lack of knowledge about the effectiveness of natural extracts in the elicitation of plant defenses.

Although several studies investigated the potentiality of extracts from plants and algae in the pre-harvest plant protection (Jayaraj et al., 2008; Ertani et al., 2013; Ahmed et al., 2016; Esserti et al., 2017; Ann Suji et al., 2019; Bajpai et al., 2019), there is a relatively little attention to their potential effectiveness in the managing of postharvest diseases.

Another interesting category of plant biostimulants is represented by inorganic salts (du Jardin, 2015). Among these, phosphites, especially potassium phosphites, stand out for their multiple actions, which include not only the mere enhancement of yield and plant defense but also a physiological function (as a P-source for plant nutrition) and a marked biocide action (Gómez-Merino and Trejo-Téllez, 2015).

The special place held by potassium phosphites in agriculture is mainly related to their effectiveness in plant disease management (Yáñez-Juárez et al., 2018), which is realized by a mode of action that can be considered the result of a combined biostimulant and biocide action (Gómez-Merino and Trejo-Téllez, 2015). Within this framework, studies on potassium phosphites described the effectiveness of these systemic products in the control of plant diseases incited by several soil- and air-borne fungal and oomycete pathogens, including *Alternaria alternata* pv. *citri*, *Colletotrichum gloeosporioides*, *Fusarium solani*, *Rhizoctonia solani*, *Penicillium* spp. and *Phytophthora* spp. (Yogev et al., 2006; Garbelotto et al., 2007; Lobato et al., 2008; MacKenzie et al., 2009; Cerioni et al., 2013; Ogoshi et al., 2013; Hunter et al., 2018; Ramallo et al., 2019; Kasuga et al., 2021), proving that their use is a practical and effective alternative to conventional fungicides for the control of plant diseases.

In accordance with the EC Regulation 2003/2003, potassium phosphites have been so far classified as fertilizers. However, during the past few years, the growing attention to the security of human health has raised concerns about phosphites. Starting from July 2022, under the new EU regulation on fertilizers (1,009/2019/EU), the use of potassium phosphites in agriculture will be allowed only after their registration as phytosanitary products, according to the 369/2013/EU. This would greatly restrict the application of phosphites even at low concentrations if they are not registered for specific uses. Within this framework, the possibility of employing natural biostimulants, either alone or in association with fungicides at reduced dosages, could represent a short-term available, and a more eco-friendly, alternative for the management of plant diseases.

**Figure 1:**
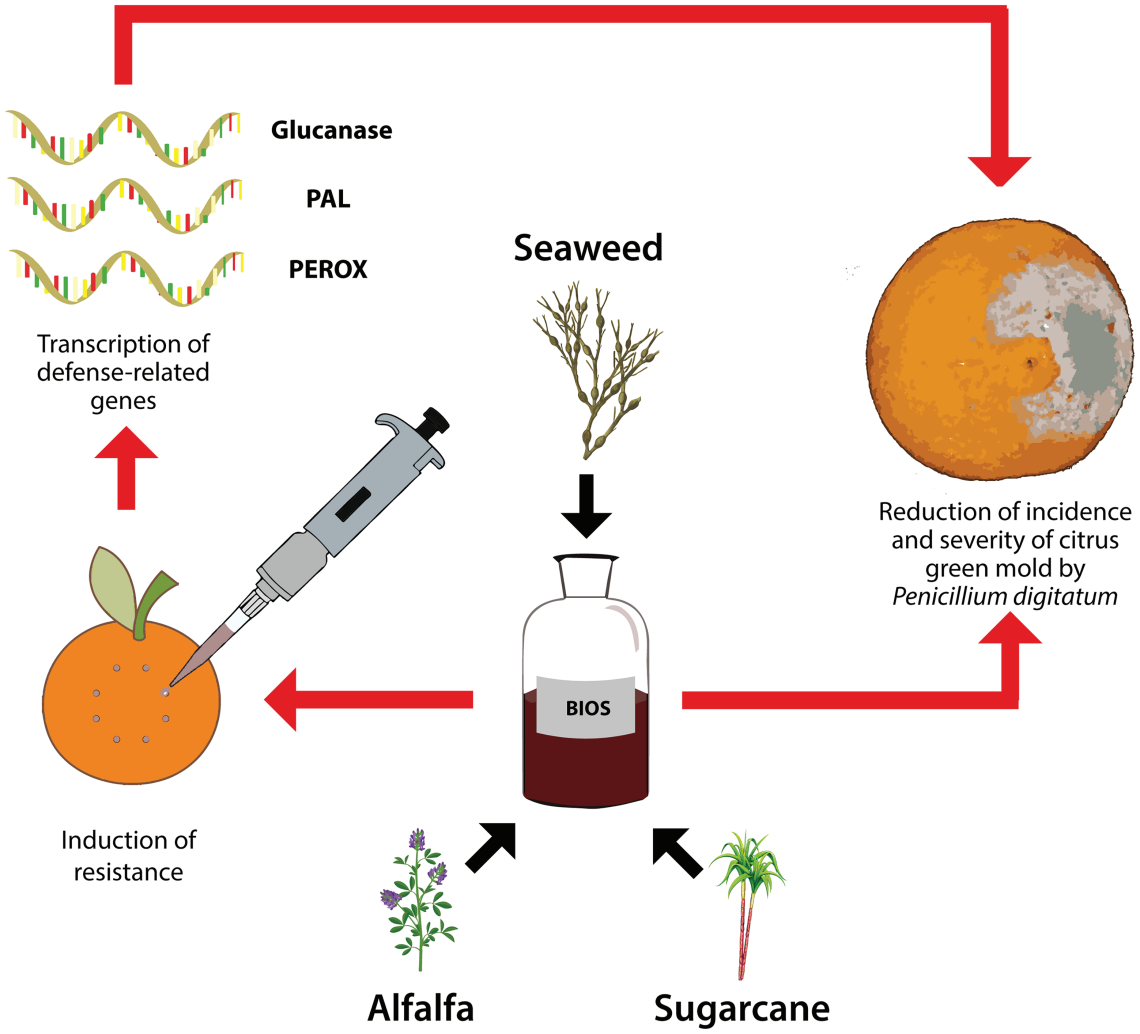
Graphical summary of the proposed experimental design for studying the effectiveness of the experimental natural biostimulant (BIOS) in triggering the transcription of plant defense-related encoding genes (β-1,3-glucanase – Glucanase, phenylalanine ammonia-lyase – PAL, and peroxidase – PEROX) and in the reduction of incidence and severity of citrus green mold by *Penicillium digitatum*.

To explore the possibility of minimizing the application of conventional fungicides to manage postharvest citrus diseases, this study investigated the effectiveness of a new natural biostimulant product, based on seaweed-extract and plant-derivatives, both alone and in mixture with a notably reduced dose of fungicide, as resistance inducer in citrus fruits to green mold incited by *P. digitatum* and as means to control the pathogen directly on infected fruits (Figure 1). The natural biostimulant was compared to the chemical biostimulant potassium phosphite and the synthetic conventional fungicide IMZ.

## Materials and Methods

### Fungal Material: Isolation and Identification

The IMZ-sensitive (MIC < 1 μml^−1^ a.i.) *P. digitatum* strain, P_digit_G.B.A., was obtained from a symptomatic fruit of *Citrus sinensis* cv. Moro nucellare lines collected from a local citrus orchard (Siracusa, Italy) managed according to the principles of organic agriculture. For the isolation, a 5 mm fragment of *Penicillium* infected citrus peel was excised from the margin of the advancing growing area, disinfected with 1% NaClO for 2 min, rinsed in sterile distilled water, and plated on potato dextrose agar (PDA) amended with streptomycin sulfate at the concentration of 0.25 g/L. After 24 h of incubation at 25°C in the dark, the growing colony was transferred on PDA. A pure culture was obtained by single-hypha transfer, and its identity was confirmed by the amplification of the Internal Transcribed Spacer (ITS) region of the nuclear ribosomal DNA (rDNA). Total DNA was extracted by using the PowerPlant® Pro DNA Isolation Kit following the instructions of the manufacturer. The amplification of the selected target region of the rDNA was performed by using the universal primer pairs ITS-1 (5'-TCCGTAGGTGAACCTGCGG-3') and ITS-4 (5'-TCCTCCGCTTATTGATATGC-3', White et al., 1990). The PCR amplification reaction was carried out in a 25 μl reaction mix containing PCR Buffer (1X), dNTP mix (0.2 mM), MgCl2 (1.5 mM), forward and reverse primers (0.5 μM each), *Taq* DNA Polymerase (1 U, Taq DNA Polymerase, recombinant - Invitrogen™), and 100 ng of DNA. The thermocycler conditions were 94°C for 3 min, followed by 35 cycles of 94°C for 30 s, 55°C for 30 s, and 72°C for 30 s and then 72°C for 10 min. A 1X TAE electrophoresis analysis in 1% agarose gel was performed to detect the obtained amplicons. The PCR product was sequenced in both directions by an external service (Macrogen, Amsterdam, The Netherlands) and sequences were analyzed by using FinchTV v.1.4.0.^1^ The identification of the *P. digitatum* P_digit_G.B.A. was finally carried out by subjecting the consensus sequence to the standard nucleotide blast (blastn) tool.^2^

### Biostimulant, Potassium Phosphite, Fungicides, and Mixtures

The substances used in this study included: (i) an experimental natural biostimulant (BIOS) based on a seaweed (*A. nodosum*) extract and plant (alfalfa and sugarcane) derivatives; (ii) a 57% (w/v) potassium phosphite (K-Phi) product; (iii) IMZ (emulsifiable concentrate formula at 50% w/v of active ingredient - a.i.; IMZ-50) and (iv) IMZ sulfate (formula at 7.5% w/v of a.i.; IMZ-S). The tested substances and relative mixtures (BIOS or K-Phi + fungicide at half of the suggested dosage), as well as their dosages, are listed in Table 1.

**Table 1:** List of substances and mixtures used in this study.

ID of treatment	Type of substance	Substance^a^/Mixture	Application dosage
01	Fungicide	IMZ-50	1,000 ppm (a.i.^∗^) - Std^∗∗^
02	Fungicide	IMZ-50	500 ppm (a.i.) - 1/2 Std
03	Fungicide	IMZ-S	375 ppm (a.i.) - Std
04	Fungicide	IMZ-S	187.5 ppm (a.i.) - 1/2 Std
05	Natural Biostimulant	BIOS	0.15%^b^
06	Biocide/biostimulant	K-Phi	0.25%^b^
07	Mixture	Mixture A (K-Phi + IMZ-50)	K-Phi: 0.25%; IMZ-50: 500 ppm (a.i.)
08	Mixture	Mixture B (BIOS + IMZ-S)	BIOS: 0.15%; IMZ-S: 187.5 ppm (a.i.)

∗ a.i., active ingredient.

∗∗ Std, suggested dosage.

a IMZ-50 (Imazalil - emulsifiable concentrate formula at 50% w/v of active ingredient – a.i.), IMZ-S (Imazalil sulfate – formula at 7.5% w/v of a.i.), BIOS (natural biostimulant formula), K-Phi (potassium phosphite formula).

b Dosage of formula.

The experiments were designed according to the current standard processing method of citrus fruits in packinghouses. Two commercial formulations of IMZ are used in packinghouses to prevent green mold in citrus fruit: a concentrated emulsion (50% a.i., IMZ-50), which usually is incorporated into fruit coatings, and a sulfate solution (7.5% a.i., IMZ-S), whose active ingredient is rapidly absorbed by the citrus peel. IMZ-S is applied exclusively as drenches or by dipping due to the lower a.i. dosage and the incompatibility of this type of formulation with fruit coating agents. However, IMZ-S alone at a half-dose is not effective in controlling *Penicillium*. On the other hand, the biostimulant has no biocide activity but was expected to have a synergistic action with the fungicide. Therefore, in this study IMZ-S (1/2 dose) was applied in combination with the biostimulant to test the efficacy of the mixture in controlling the green mold and verify whether the IMZ residue in the fruit peel was lower than that in fruits treated with the fungicide alone at the same concentration. The treatment IMZ + K-phi, already used in packinghouses, was included as a reference to directly compare a mixture of two fungicides with the mixture of a fungicide at a reduced dose and biostimulant (Table 1).

### Fruits

For each experimental assay, untreated mature orange fruits [*Citrus sinensis* (L.) Osbeck cv. Moro nucellare] collected in a local organic citrus farm were surface-sterilized with a 1% NaClO solution for 2 min, rinsed with tap water, and air-dried at room temperature.

### Evaluating Resistance Induction

To evaluate the effectiveness of the BIOS or mixture (BIOS + fungicide) to induce resistance to postharvest green mold on citrus fruits, a specific test was performed in accordance with Youssef et al. (2014). In detail, fruits were wounded with a 2-mm-diameter plastic tip (1 wound per fruit) without injuring the juice sacks below the albedo; then, 20 μl of any substance or mixture of sterile distilled water (control) were placed in the wound. Treated fruits were placed in a plastic container and incubated at 20°C for 48 h. After incubation, fruits were punctured with a 2 mm plastic tip (1 wound per fruit) approximately 10 mm away from the treatment wound; then, 10 μl of inoculum of *P. digitatum* strain, P_digit_G.B.A. (conidial suspension of concentration 10^6^ conidia/ml), was placed into the wound. The experimental assay consisted of nine treatments listed in Table 1: fruits treated with (i) sterile distilled water (controls), substance tested (ii) ID 01, (iii) ID 02, (iv) ID 03, (v) ID 04, (vi) ID 05, (vii) ID 06, (viii) ID 07, and (ix) ID 08. Each treatment had four replicates made up of four oranges each. Inoculated fruits were again placed in a plastic container and maintained at 20°C for 5 days. The disease incidence (percentage of infected fruits) and rot severity (diameter of infected fruit surface) were recorded at 3- and 5-day postinoculation (d.p.i.). The experiment was repeated once with similar results, so only results of the first experiment are reported.

### Tissue Sampling for RNA Isolation From Fruits

**Figure 2:**
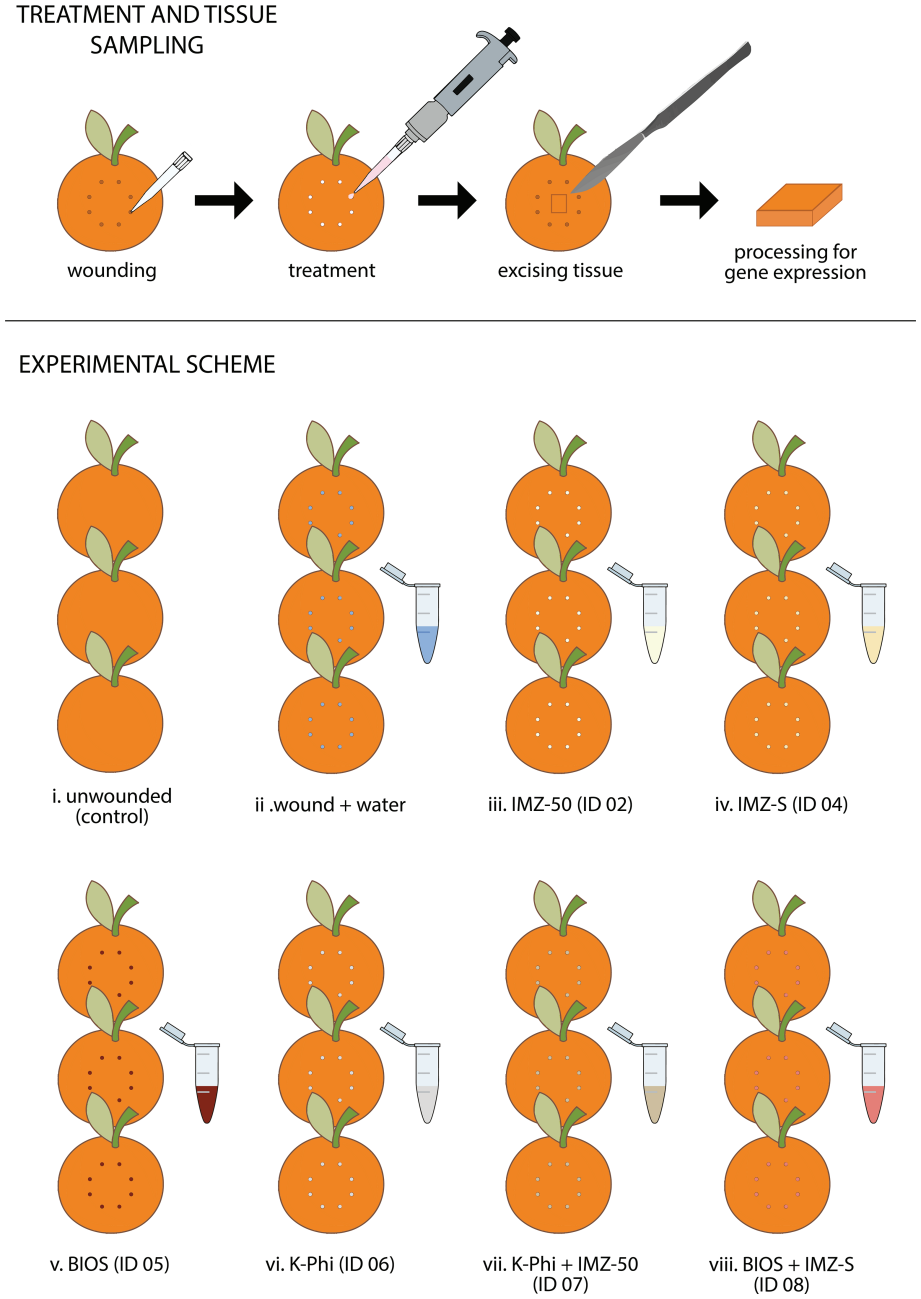
Summary of the experimental procedure and scheme employed for studying the effectiveness of test substances in triggering the differential expression of genes involved in the induction of resistance mechanisms in citrus fruits.

For the evaluation of the differential expression of genes involved in the induction of resistance in orange fruits by the different treatments, a specific test was performed (Figure 2). Fruits were individually wounded with a 2 mm plastic tip at eight points on the equatorial surface. The experimental assay consisted of the following eight treatments (see Table 1): (i) unwounded control fruits, (ii) fruits wounded and treated with sterile distilled water, fruits treated with the substance tested (iii) ID 02, (iv) ID 04, (v) ID 05, (vi) ID 06, (vii) ID 07, and (viii) ID 08. For each treatment, fruits were collected at different time intervals: 0, 24, and 48 h. For each time interval, batches of fruits were made up of three fruits. Fruits of each treatment were placed in plastic containers and maintained at 20°C for 48 h. At the time intervals, fragments of peel (5 mm ×15 mm × 2 mm) from each of three fruits of each batch were excised from the treatment site. The excised tissues were rapidly frozen in liquid nitrogen and stored at −80°C until they were used for gene expression analyses. The experiment was repeated once with similar results, so only results of the first experiment are reported.

### RNA Isolation From Orange Peels and Complementary DNA Synthesis

Total RNA was extracted by using RNeasy Plant Mini Kit (Qiagen, Netherlands) from frozen orange peels (100 mg) ground to a fine powder with liquid nitrogen, according to the protocol of the manufacturer, and treated with TURBO DNA-free™ Kit (Invitrogen, Carlsbad, CA, United States). RNA concentration was then adjusted to 200 ng/μl, and its quality was verified by performing a denaturing RNA electrophoresis gel in TAE agarose (Masek et al., 2005). Reverse transcription was performed by using a High-Capacity cDNA Reverse Transcription Kit (Applied Biosystems™, Foster City, CA, United States) according to the instructions of the manufacturer.

### Selection of Genes and Development of Specific Primers

Based on previous studies (Youssef et al., 2014), several specific primer pairs (Supplementary Table S1), targeting different genes related to the induced resistance in citrus fruits, were selected. Primers specific to the constitutively expressed housekeeping gene beta-tubulin were designed by using the Primer-BLAST NCBI tool,^3^ and their specificity was tested both by *in silico* (by using the Primer-BLAST NCBI tool) and traditional PCR.

### Quantitative Real Time-PCR Analysis of Gene Expression

Amplifications were performed by using the iCycler iQ™ Real-Time PCR Detection System (Bio-Rad, Hercules, California, USA). Reactions were performed in a total volume of 20 μl by mixing 10 ng of cDNA with 1 μl of 10 μM of each primer and 10 μl of PowerUp™ SYBR™ Green Master Mix (2X, Applied Biosystems™, Foster City, CA, United States). For each of the collected biological sample, qRT-PCR experiments were carried out in triplicate. The thermocycling conditions were 2 min at 50°C (UDG activation), 2 min at 95°C (Dual-Lock™ DNA polymerase), followed by 40 cycles of two steps: 95°C for 15 s (denaturation) and 59°C (annealing/extension) for 1 min. The quantification of gene expression relative to the unwounded control sample was carried out by using the 2^-ΔΔCt^ method (Livak and Schmittgen, 2001), where ΔΔCt = (Ct of target gene − Ct of reference gene)_sample_ − (Ct of target gene − Ct of reference gene)_calibrator_ and Ct is the threshold cycle of each transcript, defined as the point at which the amount of amplified target reaches a fixed threshold above the background fluorescence.

The PCR efficiency was checked by standard curves Ct values vs. log (cDNA dilution). Curves were constructed by serial 10-fold dilution of cDNA for each primer pair; linear equations, determination coefficients (R^2^), and reaction efficiencies are given in Supplementary Table S2.

### Evaluating *in vivo* Postinfection Activity

The *in vivo* postinfection activity of the substances tested (Table 1) was evaluated as follows: surface-sterilized orange fruits were wounded with a 2 mm-diameter plastic tip (1 wound per fruit) without injuring the juice sacks below the albedo; then, 10 μl of inoculum of *P. digitatum*, strain P_digit_G.B.A., (concentration of conidial suspension: 10^6^ conidia/ml), were placed into the wound. Inoculated fruits were placed in a plastic container and incubated at 20° C for 24 h. After incubation, fruits were immersed in an aqueous solution of each of the substances tested (Table 1) or water (controls) for 2 min, rinsed with water for 15 s, air-dried at room temperature, placed in a plastic container, and incubated at 20°C for 5 days. The experimental setup consisted of the following nine treatments (as shown in Table 1): (i) sterile distilled water (controls), substance tested (ii) ID 01, (iii) ID 02, (iv) ID 03, (v) ID 04, (vi) ID 05, (vii) ID 06, (viii) ID 07, and (ix) ID 08. Up to eight orange fruits per treatment were used. The antifungal postinfection activity of the substances tested was evaluated as rot severity (percentage of fruit surface with symptoms of rot), molding rate (percentage of fruit surface covered with mycelium), and sporulation rate (percentage of fruit surface covered by conidia) 5 days after the treatment. For each fruit, all of the abovementioned parameters were evaluated as a percentage ratio between the diameter of the area of the circular lesion (i.e., rot, mycelium, or conidia) and the total length of the fruit circumference.

### Evaluation of the Residual Concentration of Fungicide in Orange Peel

Surface-sterilized orange fruits were immersed in an aqueous solution of the substance tested (i) ID 02, (ii) ID 04, (iii) ID 07, or (iv) ID 08 (Table 1) for 2 min, rinsed with water for 15 s and air-dried at room temperature. Each treatment was represented by three batches, each consisting of about 1 kg of orange fruits.

Treated fruits were stored at 6°C for 10 days. The residual concentration of fungicides in citrus peels was then evaluated by high-performance liquid chromatography/mass spectrometry (HPLC/MS) in accordance with the method UNI EN 15662:2009 by an external service (SIALAB SRL, Italy).

The experiment was repeated one time with similar results, so only results of the first experiment are reported.

### Statistical Analyses

All data from the *in vivo* postinfection activity and resistance inducers trials, as well as ones from gene expressions, were subjected to a one-way ANOVA by using the R software.^4^ To normalize the distributions, percentage data were transformed into squareroot values, but untransformed percentages are reported in the respective graphs. The Tukey’s HSD (Honestly Significant Difference) *post hoc* test was applied to evidence statistical differences from the *in vivo* postinfection activity and the resistance inducers trials, while Dunnet’s *post hoc* test was used in the gene expression analysis. Differences at *p* ≤ 0.05 were considered significant. The mean ± SD was also included in results reported in all tables and graphs.

## Results

### Evaluation of Resistance Induction

**Figure 3:**
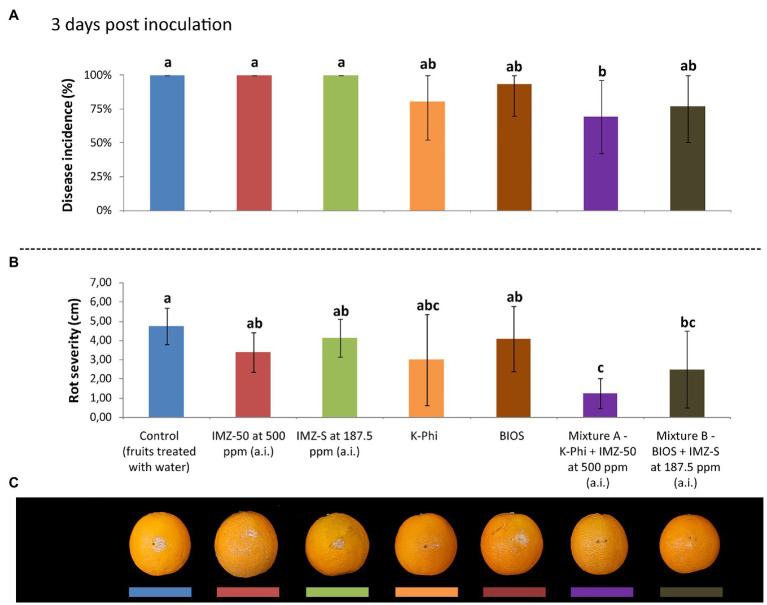
Evaluation of the induced resistance as **(A)** disease incidence (%) and **(B)** rot severity (cm), as well as observed symptoms of decay in fruits **(C)**, caused by *Penicillium digitatum* strain, P_digit_G.B.A., in *Citrus sinensis* cv. Moro nucellare orange fruits treated with water (control) or Imazalil [emulsifiable concentrate formula at 50% w/v of active ingredient (a.i.)] (IMZ-50) at 500 ppm a.i., Imazalil sulfate (formula at 7.5% w/v of a.i.; IMZ-S) at 187.5 ppm a.i., potassium phosphite (K-Phi) formula (57% w/v) at the dosage of 0.25%, natural biostimulant (BIOS) formula at the dosage of 0.15%, Mixture A (K-Phi: 0.25%; IMZ-50: 500 ppm a.i.), and Mixture B (BIOS: 0.15%; IMZ-S: 187.5 ppm a.i.) 3 days postinoculation (d.p.i.). Values sharing the same letters are not statistically different according to the Tukey’s HSD (Honestly Significant Difference) test (*p* ≤ 0.05). Bars represent SD.

**Figure 4:**
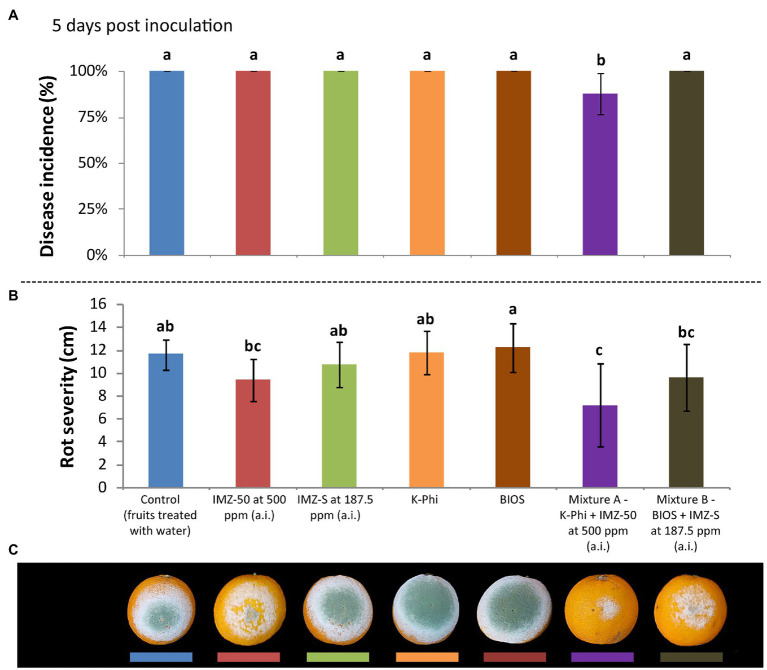
Evaluation of the induced resistance as **(A)** disease incidence (%) and **(B)** rot severity (cm), as well as observed symptoms of decay in fruits **(C)**, caused by *Penicillium digitatum* strain, P_digit_G.B.A., in *Citrus sinensis* cv. Moro nucellare orange fruits treated with water (control) or IMZ-50 (emulsifiable concentrate formula at 50% w/v of active ingredient - a.i.) at 500 ppm a.i., IMZ-S (formula at 7.5% w/v of a.i.) at 187.5 ppm a.i., K-Phi formula (57% w/v) at the dosage of 0.25%, BIOS formula at the dosage of 0.15%, Mixture A (K-Phi: 0.25%; IMZ-50: 500 ppm a.i.), and Mixture B (BIOS: 0.15%; IMZ-S: 187.5 ppm a.i.) 3 d.p.i. Values sharing the same letters are not statistically different according to the Tukey’s HSD (Honestly Significant Difference) test (*p* ≤ 0.05). Bars represent SD.

The induction of resistance was evaluated in citrus fruits treated with BIOS alone and mixed with fungicide at half of the label dose. The effects were compared with those of the treatment with the chemical inducer, K-Phi, (both alone and in mixture with half-dose of fungicide) and with the fungicides only at the same concentration of active ingredient used in the mixtures. Three d.p.i., both BIOS and K-Phi only slightly reduced the incidence and severity of rot compared to both untreated and fungicide-treated fruits (Figure 3); the observed induced resistance ended at 5 d.p.i. (Figure 4). Interestingly, among the substances tested, both mixtures showed the best effectiveness. In particular, treatment with Mixture A (K-Phi + IMZ-50) drastically reduced both the disease incidence and rot severity during all the inoculation period (Figures 3, 4), while Mixture B (BIOS + IMZ-S) maintained good effectiveness only in terms of rot severity.

### Evaluating Differences in the Expression of Induced Resistance-Related Genes in Citrus

**Figure 5:**
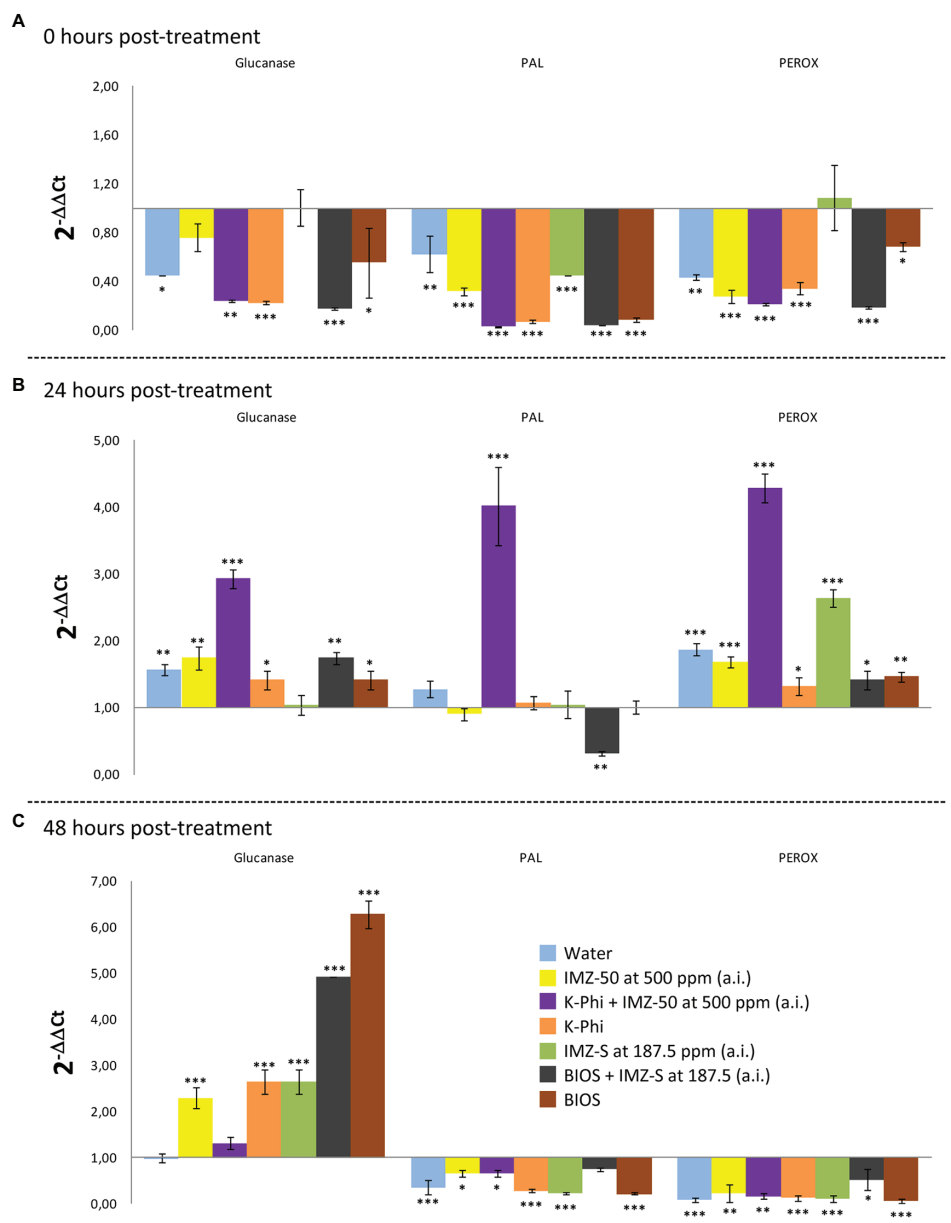
Differences in the expression levels of β-1,3-glucanases-, PAL-, and PEROX-encoding genes in fruits of sweet orange (*Citrus sinensis*) fruits cv. Moro nucellare wounded and treated with water or IMZ-50 (emulsifiable concentrate formula at 50% w/v of active ingredient - a.i.) at 500 ppm a.i., IMZ-S (formula at 7.5% w/v of a.i.) at 187.5 ppm a.i., K-Phi formula (57% w/v) at the dosage of 0.25%, BIOS formula at the dosage of 0.15%, Mixture A (K-Phi: 0.25%; IMZ-50: 500 ppm a.i.), and Mixture B (BIOS: 0.15%; IMZ-S: 187.5 ppm a.i.) at 0 **(A)**, 24 **(B)**, and 48 **(C)** h after treatment. Columns with asterisks are statistically different according to Dunnett’s test (^∗^ = *p* < 0.05, ^∗∗^ = *p* < 0.01, and ^∗∗∗^ = *p* < 0.001), as compared to unwounded fruits. Bars represent SD.

**Figure 6:**
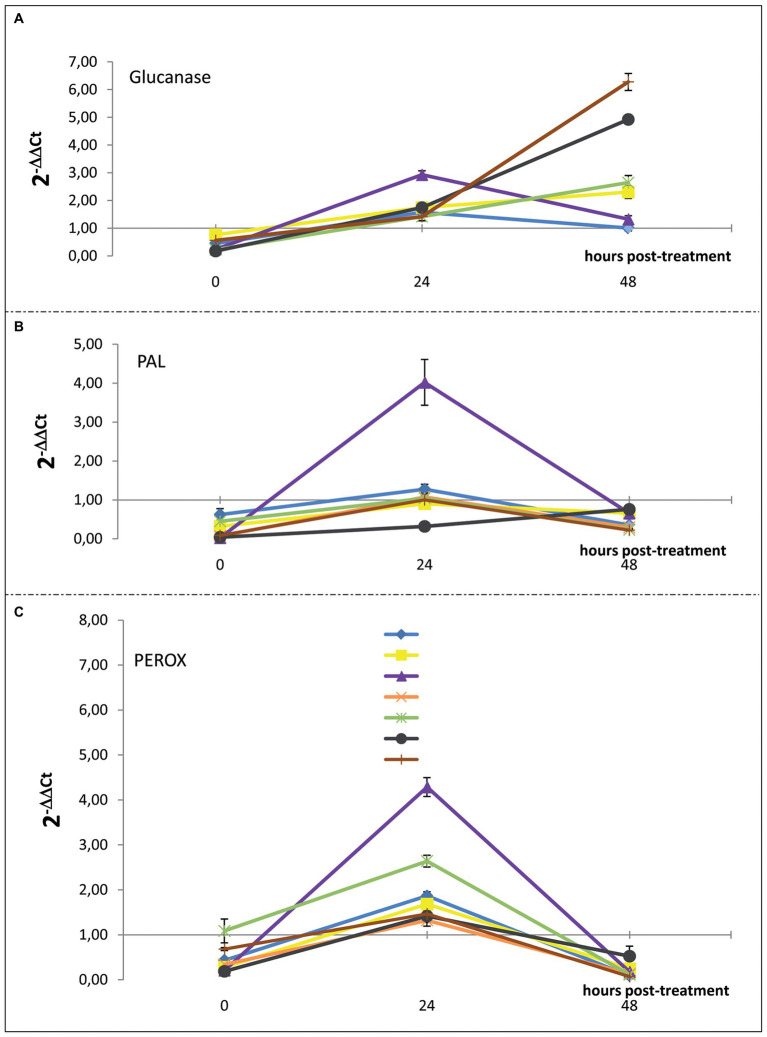
Time course of the expression levels of **(A)** β-1,3-glucanases, **(B)** PAL, and **(C)** PEROX-encoding genes in sweet orange (*Citrus sinensis*) fruits cv. Moro nucellare wounded and treated with water or IMZ-50 (emulsifiable concentrate formula at 50% w/v of active ingredient - a.i.) at 500 ppm a.i., IMZ-S (formula at 7.5% w/v of a.i.) at 187.5 ppm a.i., K-Phi formula (57% w/v) at the dosage of 0.25%, BIOS formula at the dosage of 0.15%, Mixture A (K-Phi: 0.25%; IMZ-50: 500 ppm a.i.), and Mixture B (BIOS: 0.15%; IMZ-S: 187.5 ppm a.i.). Bars represent SD.

The induced resistance in citrus fruits as a consequence of treatments with the substances tested (Table 1) was evaluated based on the expression levels of genes involved in the synthesis of enzymes related to the main plant defense pathways; these include PR proteins (i.e., β-1,3-glucanases-encoding gene), phenylalanine ammonia-lyase (i.e., PAL-encoding gene), and peroxidases (i.e., PEROX-encoding gene). The trend of the expression levels of the selected enzymes-encoding genes at 0, 24- and 48-h posttreatment is reported in Figures 5, 6.

Significant upregulation in the levels of β-1,3-glucanase, PAL-encoding, and PEROX-encoding genes in orange peels, as a consequence of the application of the substances tested, were observed 24 h after treatment. Mixture A (i.e., K-Phy + IMZ 50) determined the strongest upregulation of all three genes (Figure 5B). Only β-1,3-glucanase encoding-gene was still upregulated 48 h posttreatment (Figure 6), with the highest levels for the treatments with BIOS and Mixture B (BIOS + IMZ-S) (Figure 5C). Finally, the PAL-encoding gene was always downregulated in the treatment with Mixture B (Figure 6).

### Evaluation of *in vivo* Postinfection Activity

**Figure 7:**
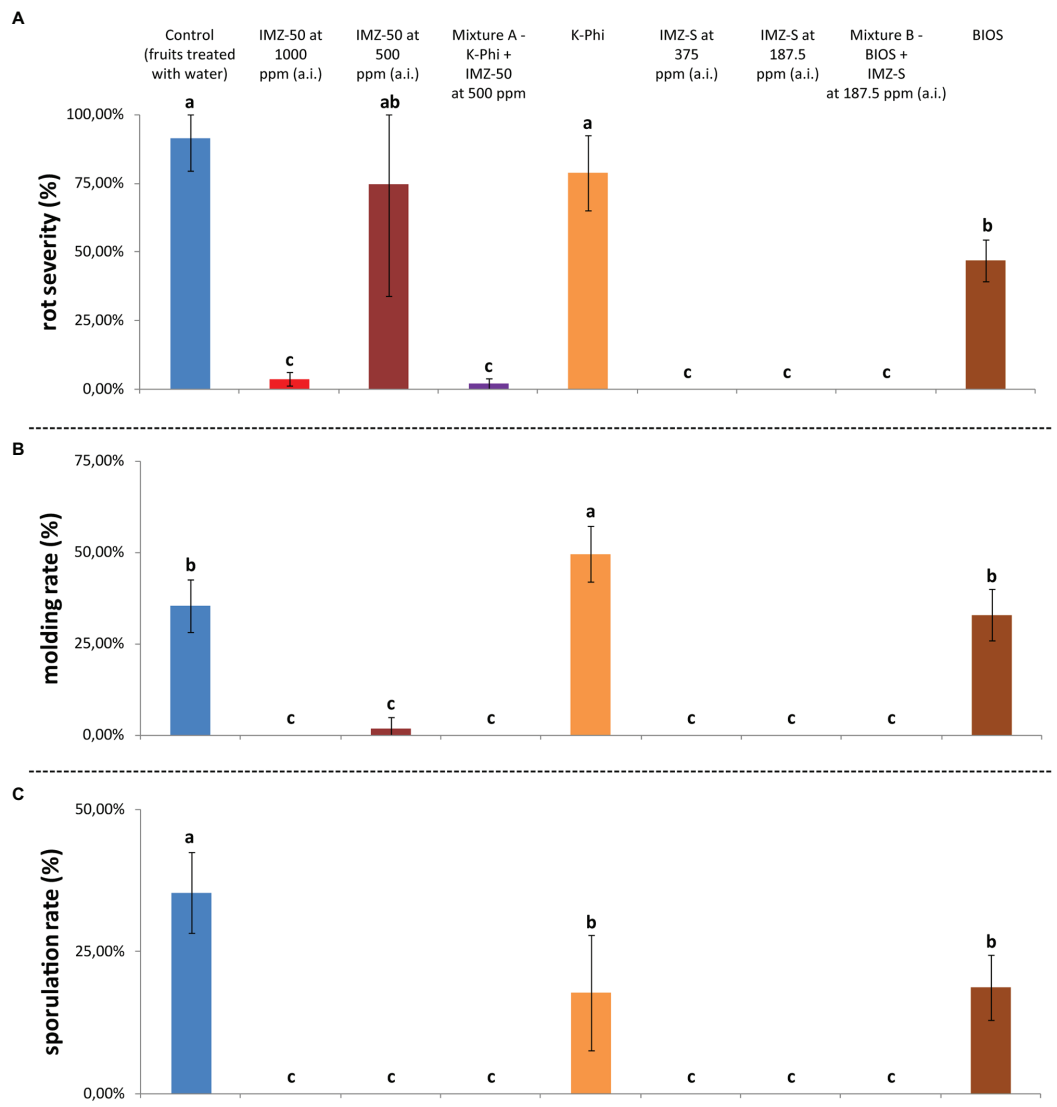
**(A)** Rot severity (%), **(B)** molding rate (%), and **(C)** sporulation rate (%) caused by *Penicillium digitatum* strain, P_digit_G.B.A., in *Citrus sinensis* cv. Moro nucellare orange fruits treated with water (control) or IMZ-50 (emulsifiable concentrate formula at 50% w/v of active ingredient - a.i.) at 1,000 ppm or 500 ppm a.i., IMZ-S (formula at 7.5% w/v of a.i.) at 375 ppm or 187.5 ppm a.i., K-Phi formula (57% w/v) at the dosage of 0.25%, BIOS formula at the dosage of 0.15%, Mixture A (K-Phi: 0.25%; IMZ-50: 500 ppm a.i.), and Mixture B (BIOS: 0.15%; IMZ-S: 187.5 ppm a.i.) 5 days after treatment. Values sharing the same letters are not statistically different according to the Tukey’s HSD (Honestly Significant Difference) test (*p* ≤ 0.05). Bars represent SD.

Results from the *in vivo* postinfection activity trial highlighted differences in the effects of the tested substances in inhibiting citrus green mold (Figure 7). When tested alone, BIOS significantly reduced both rot severity (reduction of ca. 44%) and sporulation rate (reduction of ca. 17%) compared to water-treated control fruits (Figures 7A,C); however, it did not completely control the disease. Although the exclusive application of K-Phi determined a significant reduction only of the sporulation rate (Figure 5C), when applied in combination with IMZ-50 at half of the recommended dosage (i.e., Mixture A), a drastic reduction in all evaluated parameters was reported, with a significantly improved effectiveness over the sole application of both components of Mixture A (Figure 7A).

Analogous positive effects were observed in fruits treated with Mixture B (i.e., BIOS + IMZ-S), with the advantage that, in this case, the dose of the fungicide was very low.

### Evaluation of Residual Concentration of Fungicides in Fruit Peel

To better understand the positive effects of the mixture BIOS + IMZ-S in reducing the incidence and severity of the green mold, the residual concentration of the fungicide a.i., IMZ, in Mixture B was evaluated and compared with those of fruits treated with Mixture A or individually treated with IMZ-S or IMZ-50 at the same concentration of a.i. used in the mixtures. Results revealed that the residue of Imazalil in the peel of fruits treated with Mixture A or Mixture B was significantly lower than the residue found in the peel of fruits treated with fungicides alone at the same a.i. concentration (Table 2).

**Table 2:** Residual concentration of Imazalil in citrus peel from fruits treated with IMZ-50, IMZ-S, Mixture A, and Mixture B.

Fungicide/GRAS/Mixture	Fungicide active ingredient in the application dosage (ppm of Imazalil)	Residual concentration of Imazalil (ppm) ± standard deviation
IMZ-50	500	1.780 ± 0.022
IMZ-S	187.5	0.991 ± 0.022
Mixture A (K-Phi + IMZ-50)	500	0.690 ± 0.022
Mixture B (BIOS + IMZ-S)	187.5	0.612 ± 0.022

## Discussion

This study provided evidence of the effectiveness of a natural biostimulant based on a mixture of seaweed and plant extracts, either alone or in combination with a reduced dose of the conventional synthetic fungicide IMZ, in controlling postharvest green mold of orange fruits incited by *P. digitatum*. The efficacy of this natural biostimulant was comparable to that of the chemical resistance inducer, potassium phosphite.

According to the literature, different categories of biotic and abiotic stressors and biostimulants can trigger and modulate the plant immune response (Khan et al., 2009; Deliopoulos et al., 2010; Puglisi et al., 2012; Calvo et al., 2014; du Jardin, 2015; La Spada et al., 2020, 2021; Stracquadanio et al., 2021). The effectiveness of seaweed extracts and inorganic salts in eliciting natural plant defense mechanisms for the control of fungal diseases has been widely investigated (Khan et al., 2009; Calvo et al., 2014; du Jardin, 2015).

Several studies showed the potential of seaweed products to manage preharvest plant diseases. Extracts from *A. nodosum* provided effective protection from *Alternaria radicina* and *Botrytis cinerea* foliar infections on *Daucus carota* (Jayaraj et al., 2008) by enhancing natural plant defenses. Aerial applications of extracts from *Ulva lactuca* were effective against the late blight incited by *Phytophthora infestans* (Ahmed et al., 2016). A biostimulant preparation of brown seaweeds significantly reduced powdery mildew on strawberry plants (Bajpai et al., 2019). Conversely, the effectiveness of natural products based on plant derivatives, such as pomegranate peel extracts, has been tested to control both preharvest and postharvest plant diseases, and the use of plant extracts as inducers of resistance to protect plants from disease is very promising (Pangallo et al., 2017a,b; Belgacem et al., 2019). However, this is the first time that a mixture containing seaweed extracts demonstrated to be effective in controlling postharvest diseases by eliciting the plant immune system.

Moreover, although a rich literature on the effectiveness of some categories of inorganic salts in the control of the postharvest disease has been produced (Deliopoulos et al., 2010; Cerioni et al., 2013), this study provided the first demonstration of the induction of resistance mechanisms toward *P. digitatum* infections by phosphite salts and new insight on their effects in modulating the expression of defense-related genes in healthy citrus fruits.

In this study, two different IMZ formulations, an emulsifiable concentrate (IMZ-50) and a sulfate salt (IMZ-S) have been tested. As for the sulfate salt, IMZ is very soluble in water, and in this form, it is, therefore, recommended in aqueous dipping applications. Conversely, the emulsifiable concentrate is an oily emulsion of the fungicide and is mostly recommended for incorporation in wax applications, which is a traditional postharvest treatment to extend the fruit shelf-life (Erasmus et al., 2011; Altieri et al., 2013). In the light of these observations, the biostimulant (BIOS), which was conceived to be applied by dipping and was expected to have a long-lasting resistance-induction activity, was mixed with IMZ-S to maximize the potential biostimulant effect of the mixture. Conversely, potassium phosphite was mixed with the classical oily emulsion of IMZ, a standard treatment applied in warehouses, to maximize the biocide activity of both active ingredients and prolong their effectiveness (Gómez-Merino and Trejo-Téllez, 2015).

The enhancement of defense mechanisms against *P. digitatum* infections in citrus fruits was obtained with a seaweed extract- and plant derivative-based product (BIOS), applied both alone and in combination with the fungicide IMZ-S at half of the label dose, as well as with K-Phi, both alone and in combination with a half dose of IMZ-50. The highest elicitation of defense response was provided by both mixtures, while only a slight induction of resistance was observed following the application of BIOS and K-Phi, singularly. Mixture B (BIOS + IMZ-S) determined a significant reduction of disease symptoms but was less effective than Mixture A (K-Phi + IMZ-50), which sharply reduced both the incidence and severity of rot compared to untreated fruits and to fruits treated only with the fungicide. Both mixtures showed a significant activity up to 5 d.p.i. There are not many examples of the postinfection protective effects of the combined application of biostimulants and fungicides in the management of plant diseases, and this is the first study reporting the effectiveness of a seaweed extract- and plant derivatives-based product, either alone or with half of a dose of IMZ, in reducing citrus fruit infections by *P. digitatum*. Results of this study confirm that the addition of a biostimulant improves the effectiveness of IMZ against citrus fruit mold caused by *Penicillium* spp., making it possible to reduce the dosage of fungicide (Cerioni et al., 2013). In a study on potato late blight incited by *P. infestans*, potassium phosphite in combination with reduced doses of fungicides provided the same level of protection as treatments with the full recommended dose of fungicides (Liljeroth et al., 2016). A similar synergistic effect was observed in leaf infections of carrot seedlings by *A. radicina* and *B. cinerea*, by alternating applications of seaweed extracts and fungicides (Jayaraj et al., 2008).

The results of the present study indicate that the synergistic effect of the mixture biostimulant/fungicide in controlling the green mold of citrus fruits was due to the stimulation of the plant immune system. A significant transcriptional upregulation of β-1,3-glucanase-, PEROX-, and PAL-encoding genes was observed in fruits treated with the mixtures natural biostimulant/IMZ-S and K-Phi/IMZ-50 24 h after treatment. Consistently with the effectiveness in preventing the infections by *P. digitatum*, the highest gene expression levels were observed in fruits treated with the mixture K-Phi + IMZ-50 (Mixture A).

In particular, in fruits treated with Mixture A, PAL-encoding gene transcripts by far exceeded those of wounded fruits and of fruits treated individually with the fungicide, the chemical inducer, and the natural biostimulant. The PAL metabolism in citrus peel plays a key role in resistance mechanisms to the pathogens, since it is involved in the synthesis of protective molecules, including flavonoids (Del Río et al., 1998; Arcas et al., 2000), p-coumaric acid derivatives (Kim et al., 1991; Afek et al., 1999), and antifungal compounds, such as the phytoalexins scoparone and scopoletin (Youssef et al., 2014), whose high concentrations have been associated to the response to infections by *P. digitatum* (Kim et al., 1991; Droby et al., 2002; Ballester et al., 2010). In a similar experiment, upregulation of PAL was induced in citrus fruits treated with sodium carbonate and bicarbonate (Youssef et al., 2014); however, differently from the present study, the elicitation was mainly considered as a consequence of wound. In the present study, the elicitation effect of the PAL-encoding gene by Mixture A was only transient, as the gene was slightly downregulated 48 h after the treatment. The same result was obtained by other authors for the sodium carbonate (Youssef et al., 2014).

Mixture A also strongly elicited the transcription of the PEROX-encoding gene only at 24 h posttreatment. Since it is known that both PAL and peroxidase enzymes are involved in plant protection mechanisms from biotic and abiotic stresses (Almagro et al., 2009), it can be inferred that they both contributed to the effectiveness of this mixture in controlling green mold. High levels of peroxidases were reported from the citrus peel in response to *P. digitatum* infection (Ballester et al., 2006), indicating a primary role of this metabolite in plant-pathogen interaction. Being involved in the metabolism of lignin and ROS (Almagro et al., 2009), the synthesis of peroxidases implies the reduction of susceptibility of plant tissues to fungal infections.

By contrast, the treatment with Mixture A did not considerably elicit the upregulation of β-1,3-glucanase-encoding gene compared to both non-treated, wounded fruits and fruits treated singularly with K-Phi or IMZ-50. Furthermore, the β-1,3-glucanase, an enzyme that degrades the β-1,3-glucane, one of the main components of the cell wall of fungi (Youssef et al., 2014), was the only still upregulated gene 48 h after treatment and, unexpectedly, was normally expressed in both wounded and Mixture A-treated fruits and, by contrast, was dramatically upregulated in fruits treated with BIOS both alone and mixed with the fungicide (Mixture B). This is in agreement with a study reporting the activity of β-1,3-glucanase in *D. carota* seedlings treated with a seaweed extract (Jayaraj et al., 2008), making it possible to speculate that seaweed extract-based biostimulants elicit this particular plant defense pathway, thus providing prolonged protection against fungal infections.

Overall, results from gene expression suggest that the treatment with mixture K-Phi + IMZ-50 (Mixture A) exert strong protection from infections, including those caused by *P. digitatum*; it is early but transient while the resistance induced by the mixture of natural biostimulant + IMZ-S (Mixture B) is delayed, but long-lasting.

The inhibitory effects on citrus green mold make it possible to speculate that the tested natural biostimulant could not only act as a directed inductor of a defense response but also, like other natural compounds, as a resistance inductor by priming mechanisms (Aranega-Bou et al., 2014). In particular, it can be hypothesized that this biostimulant triggered permanent changes of plant response at the physiological, transcriptional, metabolic, and epigenetic levels, thus determining an enhancement of resistance and/or stress tolerance (Mauch-Mani et al., 2017). Further research is needed to verify this hypothesis.

Finally, results of this study demonstrated that IMZ-S at half of the label dose mixed with the seaweed extract- and plant extract-based biostimulant was as effective as IMZ-S at the full standard dose in preventing citrus fruit infections by green mold, suggesting that this natural biostimulant could be used in an integrated disease management strategy to reduce the number of synthetic fungicides applied in citrus packinghouses. Very interestingly and quite surprisingly, the mixture of natural biostimulant and IMZ-S (Mixture B) significantly reduced the number of synthetic fungicide residues in the fruit peel compared to the fruits treated with the fungicides alone at the same dose as well as the fruits treated with a Mixture A (K-Phi + fungicide). The final concentration of IMZ a.i. in the peel of orange fruits treated with the mixture of natural biostimulant plus fungicide was 0.612 ppm, far below the threshold value indicated by the European Union, which is 4 ppm for grapefruits and oranges and 5 ppm for lemons, limes, and mandarins (Commission Regulation EU 2019/1582). The mechanisms determining a dramatic reduction of fungicide residues in fruits treated also with the natural biostimulant deserve to be further investigated as they have toxicological and food safety practical implications.

## Conclusion

The effectiveness showed by a biostimulant based on a mixture of seaweed and plant extracts in preventing green mold incited by *P. digitatum* and the synergistic effect of this new product with the fungicide IMZ offer new practical and readily available alternatives to the conventional management of postharvest rot of orange fruit, based exclusively on the use of synthetic fungicides.

The short-term objective is to reduce the number of toxic residues in the fruit peel. New insights resulting from the study of the genetic mechanisms involved in the activation and modulation of plant immune response by both this natural biostimulant and the chemical resistance inducer, potassium phosphite, are the premise for a more general objective, which is to seek more eco-friendly solutions to manage postharvest fungal diseases of citrus fruits.

## Data Availability Statement

The original contributions presented in the study are included in the article/Supplementary Material, further inquiries can be directed to the corresponding authors.

## Author Contributions

SOC, FLS, MC, and AP conceptualized the study, analyzed the results, and reviewed and edited the draft. FLS and FA did the investigation and formal analysis and performed the experiments. SOC, MC, and AP were responsible for funding acquisition and supervised the study. FLS wrote the original draft. All authors contributed to the article and approved the submitted version.

### Conflict of Interest

MC was employed by the company Decco Italia S.R.L.

The remaining authors declare that the research was conducted in the absence of any commercial or financial relationships that could be construed as a potential conflict of interest.

## Footnotes

^1^https://digitalworldbiology.com/FinchTV

^2^https://blast.ncbi.nlm.nih.gov/Blast.cgi?PROGRAM=blastn>PAGE_TYPE=BlastSearch>LINK_LOC=blasthome

^3^https://www.ncbi.nlm.nih.gov/tools/primer-blast/

^4^https://www.r-project.org/
