# Supplementary material for: Natural Biostimulants Elicit Plant Immune System in an Integrated Management Strategy of the Postharvest Green Mold of Orange Fruits Incited by Penicillium digitatum
Source: Front Plant Sci. 2021 Jun 15;12:684722. doi: 10.3389/fpls.2021.684722 (PMC8239386; doi:10.3389/fpls.2021.684722)
Supplement: Supplementary file 1 [file Data_Sheet_1.PDF]

Table S1. Gene-specific primers used in quantitative reverse transcription real-time polymerase chain reaction (qRT-PCR).

Target gene (gene symbol)	Genebank accession n.	Gene description	Primers	Melting temperature (°C)	Amplicone size (bp)
LOC102631140 (Beta tube)	XM_006473602	*Citrus sinensis* beta-tubulin (housekeeping gene)	**Csis_Betatub_For** 5'-TCTGATCTCTGCAACTATGAGTG -3'	57.74	198
			**Csis_Betatub_Rev** 5'-ATTTGCTGGGTCAGCTCTGG -3'	60.32	
LOC18032133 (beta-1,3-glucanase)	XM_024191934	*Citrus sinensis* beta-1,3-glucanase	**Gluc 2R** 5'-ATGGCGTCAAAAAGACTTCG-3'	57.03	164
			**Gluc 2F** 5'-ATTCGCTTCCTCAACGAAAA-3'	55.98	
LOC102620464 (PAL)	XM_006481431	*Citrus sinensis* phenylalanine ammonia-lyase-like	**PAL 1F** 5'-GCTCATGTTTGCCCAATTTT-3'	55.68	153
			**PAL 2R** 5'-AGAAATTGGAGCTCGGAACA-3'	57.14	
gpx1 (POX)	AJ582678.1	*Citrus sinensis* gpx1 gene for phospholipid hydroperoxide glutathione peroxidase	**POX 1R** 5'-TCTGCAAGGGGGTAACAAAC-3'	58.01	240
			**POX 2F** 5'-GCAAAGGTGGACTTTTTGGA-3'	56.73	

Table S2. Linear equations, determination coefficients (R^2) and reaction efficiencies obtained by plotting cDNA concentrations (log ng) and Ct values experimentally achieved by real-time PCR for each gene evaluated in this study.

Gene symbol	Linear equation	R^2	Reaction efficiency (%)
Beta tube	y = -3.3157x + 24,522	0.998	100.26
beta-1,3-glucanase	y = -3.3326x + 24,362	0.9996	99.56
PAL	y = -3.2999x + 23,029	0.9994	100.93
POX	y = -3.3245x + 25,482	0.9992	99.90
